# J-shaped association between metabolic score for visceral fat and albuminuria risk: a population-based study

**DOI:** 10.3389/fendo.2025.1649521

**Published:** 2025-10-01

**Authors:** Yuren Zhang, Jinting Xu, Yiping Zhang, Yinan Chen, Qiaolan Liu

**Affiliations:** Department of Endocrinology & Metabolism, Jinjiang Municipal Hospital, Shanghai Sixth People’s Hospital Fujian, Quanzhou, Fujian, China

**Keywords:** albuminuria, urinary albumin-to-creatinine ratio, metabolic score for visceral fat, NHANES, population-based study

## Abstract

**Objective:**

Metabolic Score for Visceral Fat (METS-VF) represents a novel metric for assessing visceral fat and its associated cardiometabolic risks. This study evaluated the relationship between METS-VF and the prevalence of albuminuria among U.S. adults.

**Methods:**

A cross-sectional study enrolled participants aged 20 years and older from the National Health and Nutrition Examination Surveys (NHANES) between 1999 and 2018. Albuminuria was identified as a urinary albumin-to-creatinine ratio (UACR) of 30 mg/g or higher. The Metabolic Score for Visceral Fat (METS-VF) was assessed using the Metabolic Score for Insulin Resistance (METS-IR), waist-to-height ratio (WHtR), age, and sex. The association between METS-VF and the risk of albuminuria was explored.

**Results:**

Among the 22514 adult participants, the albuminuria group exhibited higher METS-VF levels compared to the non-albuminuria group. Furthermore, the prevalence of albuminuria increased progressively with rising METS-VF levels. Adjusted multivariable logistic regression analysis revealed a significant association between METS-VF and the risk of albuminuria (OR = 1.406, 95%CI:1.243-1.590, P<0.001). Restricted cubic spline analysis demonstrated a J-shaped dose-response relationship, with a threshold value of 6.128. Mediation analysis further identified hemoglobin A1c (HbA1c), blood pressure, oxidative stress, and inflammation as partial mediators of this association.

**Conclusion:**

METS-VF may act as a useful epidemiological indicator for assessing visceral fat’s role in albuminuria risk among U.S. adults. Additional large-scale prospective research is necessary for confirmation.

## 1 Introduction

Representing a major public health issue, chronic kidney disease (CKD) is a progressive condition affecting roughly 10% of people globally (1, 2). Albuminuria, a hallmark diagnostic feature of CKD, arises from pathological increases in urinary albumin excretion secondary to glomerular damage. This manifestation is particularly prominent in diabetic nephropathy and hypertensive nephropathy (3–5). Importantly, during early glomerular injury, conventional urine protein assays may yield normal results despite elevated albuminuria levels, which escalate progressively with disease severity (6). Additionally, substantial evidence confirms that albuminuria functions not only as a sensitive biomarker for incipient renal injury and vascular endothelial dysfunction, but also as an independent prognostic indicator for CKD progression, cardiovascular events, and all-cause mortality (7–9).

Obesity exhibits substantial pathophysiological heterogeneity, with the distribution of adipose tissue playing a critical role in modulating associated health risks (10, 11). Visceral adipose tissue (VAT), a metabolically active intra-abdominal fat depot surrounding internal organs, poses a significantly greater cardiometabolic risk compared to subcutaneous adipose tissue (12, 13). To enable more precise quantification of the metabolic effects of visceral adiposity, researchers have developed the Metabolic Score for Visceral Fat (METS-VF) (14). This innovative composite metric combines key metabolic parameters—including the Metabolic Score for Insulin Resistance (METS-IR), waist-to-height ratio (WHtR), age, and sex—to serve as a robust clinical tool for evaluating visceral fat burden (14). Compared to alternative surrogate markers, METS-VF demonstrates superior capacity to discriminate visceral adiposity and its associated cardiometabolic risks (14, 15). Consistent findings from epidemiological studies link elevated METS-VF to increased risks of diabetes mellitus, hypertension, nonalcoholic fatty liver disease (NAFLD), cardiovascular diseases (CVDs), and mortality (15–19).

Emerging evidence highlights VAT as a key modifiable factor influencing the risk of albuminuria (20, 21). However, the specific relationship between METS-VF and albuminuria risk remains uncharacterized. To address this critical knowledge gap, we employed the National Health and Nutrition Examination Surveys (NHANES) database to systematically evaluate the relationship between METS-VF and albuminuria prevalence in a nationally representative cohort.

## 2 Materials and methods

### 2.1 Data source

**Figure 1:**
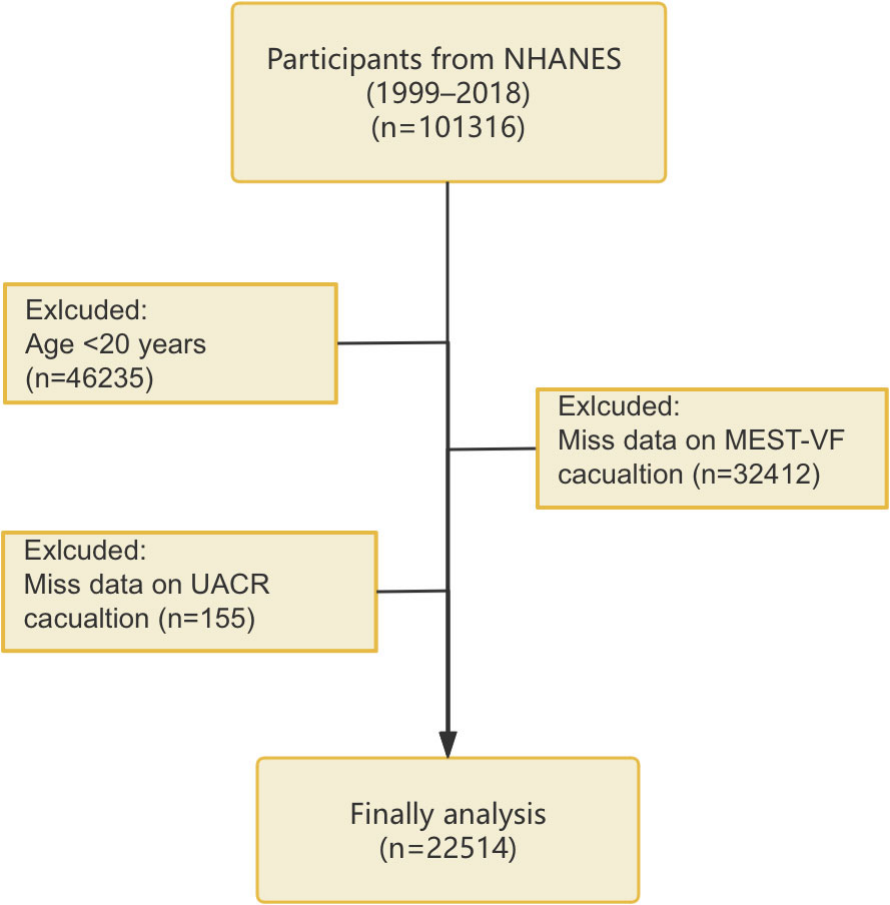
Flowchart of participant screening process.

Our study population was obtained from the NHANES database. NHANES, carried out by the National Center for Health Statistics (NCHS) under the Centers for Disease Control and Prevention (CDC) adopts a stratified, multistage, randomized sampling framework to represent the U.S. population at a national level. Participants underwent questionnaires, clinical examinations, and laboratory analyses. The study protocol was approved by the NCHS Ethics Review Board, and all participants gave written informed consent (https://www.cdc.gov/nchs/nhanes/about/erb.html). The analysis used data from 10 NHANES cycles conducted between 1999–2000 and 2017-2018. Exclusions applied to those under 20 years old or missing METS-VF or the urinary albumin-to-creatinine ratio (UACR) data, resulting in 22514 eligible participants (**Figure 1**).

### 2.2 Exposure and outcome

METS-VF was the exposure variable in this study. METS-VF= 4.466 + 0.011 × [Ln (METS-IR)] ³ + 3.239 × [Ln (WHtR)] ³ + 0.319 × (sex) + 0.594 × [Ln (age)] (male=1, female=0) (14). METS-IR= Ln [(2 × fasting plasma glucose) + (triglycerides)] × (body mass index)/[Ln (high-density lipoprotein cholesterol)] (22). On the other hand, albuminuria (UACR ≥30 mg/g) was the outcome variable in this study. UACR was calculated as urinary albumin divided by urinary creatinine. Urinary albumin was measured using solid-phase fluorescence immunoassay, and urinary creatinine was assessed via the modified Jaffe kinetic method. Detailed measurement methods are provided at: https://wwwn.cdc.gov/nchs/nhanes.

### 2.3 Covariates

Potential covariates included age, sex, ethnicity, marital status, poverty-income ratio (PIR), education level, smoking history, hypertension, diabetes, CVDs, body mass index (BMI), waist circumference (WC), height, systolic blood pressure (SBP), diastolic blood pressure (DBP), fasting plasma glucose (FPG), hemoglobin A1c (HbA1c), triglycerides (TG), total cholesterol (TC), high-density lipoprotein cholesterol (HDL-c), low-density lipoprotein cholesterol (LDL-c), and the estimated glomerular filtration rate (eGFR). Ethnicity categories included Mexican American, Other Hispanic, Non-Hispanic White, Non-Hispanic Black, and individuals of Other Race. Education was categorized as less than high school, high school, or college or above. Smoking status included current and former smokers. BMI is calculated as weight (kg)/height² (m²) and classified as <25 kg/m^2^, 25–30 kg/m^2^, and ≥30 kg/m^2^. The eGFR was estimated using the Chronic Kidney Disease Epidemiology Collaboration equation (23). Hypertension was defined as SBP ≥140 mmHg, DBP ≥90 mmHg, self-reported history, or antihypertensive medication use. Diabetes was defined as FPG ≥126 mg/dL, HbA1c ≥6.5%, self-reported history, or hypoglycemic medication use. Self-reported heart attack, stroke, heart failure, coronary artery disease, or angina was used to determine the presence of CVDs. Detailed measurements are provided at: https://wwwn.cdc.gov/nchs/nhanes.

### 2.4 Statistical analysis

To align with the guidelines, the analysis utilized descriptive statistics weighted by population(https://wwwn.cdc.gov/nchs/nhanes/analyticguidelines.aspx). Continuous variables were reported as median (interquartile range), and categorical variables as counts (weighted percentages). Missing data were assumed to be missing at random and imputed using the random forest algorithm. Group comparisons used Kruskal-Wallis and chi-square tests. Logistic regression models assessed the association between METS-VF and albuminuria risk, with three adjustment levels: Model 1 (unadjusted), Model 2 (adjusted for age group (<60/≥60 years), sex, ethnicity, marital status, PIR, education level, and smoking history), and Model 3 (Model 2 + hypertension, diabetes, CVDs, BMI group (<25/25-30/≥30kg/m^2^), TC, LDL-c, and eGFR). Restricted cubic splines (RCS) were employed to examine potential nonlinearity. The optimal threshold value was determined through maximum likelihood estimation. Risk differentials across the identified threshold were then quantified using a segmented regression approach. Receiver operating characteristic (ROC) curves were conducted with the `pROC` package and decision curve analyses (DCA) with the `rmda` package in R to compare METS−VF with other indicators for classification accuracy and clinical utility, based on default parameters. Stratified analyses were conducted by age, sex, ethnicity, BMI, hypertension, diabetes, CVDs, and eGFR. Mediation analyses using the Sobel test were conducted to evaluate whether HbA1c, blood pressure (SBP, DBP), oxidative stress (gamma−glutamyl transferase [GGT], serum uric acid [SUA]), and inflammation (white blood cell count [WBC], systemic immune−inflammation index [SII], neutrophil−to−lymphocyte ratio [NLR]) mediated the relationship between METS-VF and albuminuria risk (24). Statistical analyses used R software (version 4.2.0), with P<0.05 considered significant.

## 3 Results

### 3.1 Baseline characteristics

**Figure 2:**
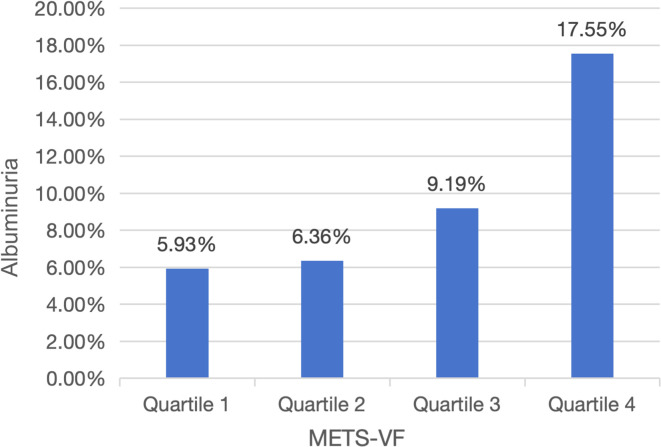
Changes in albuminuria across METS−VF quartiles.

The analysis included 22514 adults (median age: 49 years; interquartile range: 34–64 years) with ethnic distribution: 4057 (weighted: 8.50%) Mexican American, 1918 (weighted: 5.43%) Other Hispanic, 10042 (weighted: 68.25%) Non-Hispanic White, 4429 (weighted: 10.46%) Non-Hispanic Black, and 2068 (weighted: 7.37%) Other Race (**Table 1**). Albuminuria patients tended to be older and exhibited unfavorable socioeconomic and lifestyle characteristics, including lower PIR, less education, being unmarried, and smoking (P<0.001). They also had shorter stature, lower HDL-c, LDL-c, and eGFR, but higher BMI, WC, SBP, DBP, FPG, HbA1c, TG, and METS-VF levels (P<0.001). Additionally, they showed a higher prevalence of hypertension, diabetes, and CVDs (P<0.001). Participants stratified by METS-VF quartiles (Q1–Q4) revealed that higher quartile groups were older, predominantly male, and more likely to have lower PIR, less education, be married, and smoke (**Table 2**) (P<0.001). They also had higher BMI, WC, FPG, HbA1c, SBP, DBP, TG, TC, and LDL-c levels, lower HDL-c and eGFR levels, and a significantly higher prevalence of hypertension, diabetes, and CVDs compared to Q1 (P<0.001). Notably, albuminuria prevalence increased with METS-VF levels (P<0.001) (**Figure 2**).

**Table 1:** Baseline characteristics stratified by albuminuria status.

Characteristics	Overall (n=22514)	Non-albuminuria (n=19703)	Albuminuria (n=2811)	P value
Age (years)	49.00 (34.00-64.00)	47.00 (33.00-62.00)	62.00 (46.00-74.00)	<0.001
Sex, n%				0.659
Female	11621 (51.29%)	10181 (50.95%)	1440 (54.62%)	
Male	10893 (48.71%)	9522 (49.05%)	1371 (45.38%)	
Ethnicity, n%				<0.001
Mexican American	4057 (8.50%)	3481 (8.26%)	576 (10.71%)	
Other Hispanic	1918 (5.43%)	1699 (5.41%)	219 (5.55%)	
Non-Hispanic White	10042 (68.25%)	8920 (68.96%)	1122 (61.46%)	
Non-Hispanic Black	4429 (10.46%)	3777 (10.07%)	652 (14.17%)	
Other Race	2068 (7.37%)	1826 (7.30%)	242 (8.11%)	
PIR	2.19 (1.21-3.92)	2.25 (1.23-4.04)	1.81 (1.08-3.12)	<0.001
Educational level, n%				<0.001
Less than High school	6066 (16.74%)	5026 (15.87%)	1040 (25.13%)	
High school	5157 (23.52%)	4488 (23.11%)	669 (27.52%)	
Some college or above	11291 (59.73%)	10189 (61.03%)	1102 (47.35%)	
Married, n%	12180 (56.78%)	10769 (57.37%)	1411 (51.12%)	<0.001
Smoking history, n%	10370 (46.30%)	8949 (45.87%)	1421 (50.43%)	<0.001
Hypertension, n%	8989 (36.17%)	7105 (33.59%)	1884 (60.85%)	<0.001
Diabetes, n%	3892 (13.79%)	2706 (11.32%)	1186 (37.52%)	<0.001
CVDs, n%	2369 (8.67%)	1685 (7.28%)	684 (21.93%)	<0.001
BMI (kg/m^2^)	27.80 (24.24-32.15)	27.66 (24.18-31.92)	28.94 (24.83-33.95)	<0.001
WC (cm)	97.40 (87.50-108.00)	96.90 (87.00-107.40)	101.80 (92.00-113.20)	<0.001
Height (cm)	167.00 (160.00-174.60)	167.20 (160.30-174.80)	165.30 (157.80-172.60)	<0.001
FPG (mg/dL)	99.10 (92.00-110.00)	99.00 (91.60-108.00)	108.50 (96.10-138.00)	<0.001
HbA1c (%)	5.50 (5.20-5.80)	5.40 (5.20-5.80)	5.80 (5.40-6.80)	<0.001
SBP (mmHg)	120.62 (111.33-132.25)	119.33 (110.67-130.00)	134.00 (120.67-148.00)	<0.001
DBP (mmHg)	69.96 (63.33-76.00)	69.63 (63.33-76.00)	70.67 (62.90-78.00)	<0.001
TG (mg/dL)	107.00 (73.00-159.00)	104.00 (72.00-155.00)	122.00 (84.00-186.00)	<0.001
TC (mg/dL)	192.00 (166.00-221.00)	192.00 (167.00-221.00)	191.00 (164.00-222.00)	0.401
HDL-c (mg/dL)	50.00 (42.00-63.00)	51.00 (42.00-63.00)	49.00 (41.00-61.00)	<0.001
LDL-c (mg/dL)	113.00 (91.00-138.00)	114.00 (91.51-138.00)	110.00 (87.00-137.00)	0.001
eGFR (ml/min/1.73m^2^)	97.86 (81.13-113.43)	99.03 (83.35-114.28)	86.12 (62.52-105.85)	<0.001
METS-VF	6.97 (6.45-7.36)	6.93 (6.40-7.31)	7.28 (6.87-7.59)	<0.001

PIR, Poverty-Income Ratio; CVDs, Cardiovascular Diseases; BMI, Body Mass Index; WC, Waist Circumference; FPG, Fasting Plasma Glucose; HbA1c, Hemoglobin A1c; SBP, Systolic Blood Pressure; DBP, Diastolic Blood Pressure; TG; Triglycerides; TC, Total Cholesterol, HDL-c, High-Density Lipoprotein Cholesterol, LDL-c, Low-Density Lipoprotein Cholesterol; eGFR, Estimated Glomerular Filtration Rate, METS-VF, Metabolic Score for Visceral Fat.

**Table 2:** Baseline characteristics by METS-VF quartiles (Q1-Q4).

Characteristics	Quartile 1	Quartile 2	Quartile 3	Quartile 4	P value
Age (years)	32.00 (25.00-44.00)	43.00 (32.00-58.00)	53.00 (41.00-66.00)	64.00 (53.00-73.00)	<0.001
Sex, n%					<0.001
Female	3447 (62.19%)	3268 (56.01%)	2874 (48.22%)	2032 (35.99%)	
Male	2182 (37.81%)	2360 (43.99%)	2754 (51.78%)	3597 (64.01%)	
Ethnicity, n%					<0.001
Mexican American	741 (6.67%)	1096 (9.44%)	1208 (10.57%)	1012 (7.39%)	
Other Hispanic	394 (5.09%)	506 (6.29%)	506 (5.47%)	512 (4.78%)	
Non-Hispanic White	2524 (66.82%)	2371 (65.90%)	2365 (67.02%)	2782 (73.98%)	
Non-Hispanic Black	1185 (11.16%)	1057 (10.11%)	1115 (10.89%)	1072 (9.54%)	
Other Race	785 (10.27%)	598 (8.25%)	434 (6.04%)	251 (4.31%)	
PIR	2.29 (1.21-4.16)	2.27 (1.22-4.07)	2.15 (1.20-3.88)	2.08 (1.20-3.59)	<0.001
Educational level, n%					<0.001
Less than High school	1099 (13.21%)	1405 (15.50%)	1684 (18.92%)	1878 (20.11%)	
High school	1196 (20.18%)	1292 (23.42%)	1340 (25.09%)	1329 (26.02%)	
Some college or above	3334 (66.62%)	2931 (61.08%)	2604 (55.99%)	2422 (53.87%)	
Married, n%	2441 (45.62%)	3163 (59.46%)	3240 (61.70%)	3336 (61.90%)	<0.001
Smoking history, n%	2332 (42.22%)	2352 (43.34%)	2601 (47.16%)	3085 (53.74%)	<0.001
Hypertension, n%	802 (12.85%)	1681 (28.10%)	2700 (44.28%)	3806 (64.93%)	<0.001
Diabetes, n%	137 (1.95%)	459 (6.20%)	1086 (15.10%)	2210 (35.43%)	<0.001
CVDs, n%	149 (2.15%)	333 (5.06%)	623 (9.49%)	1264 (19.81%)	<0.001
BMI (kg/m^2^)	22.60 (20.70-24.57)	26.88 (24.60-29.40)	29.40 (26.85-32.90)	33.69 (30.20-38.48)	<0.001
WC (cm)	81.00 (76.10-86.00)	93.60 (89.10-98.70)	101.60 (96.70-107.60)	114.10 (107.20-123.20)	<0.001
Height (cm)	167.20 (160.80-174.60)	166.10 (159.40-174.10)	166.20 (159.10-174.20)	168.30 (160.50-175.20)	<0.001
FPG (mg/dL)	92.60 (87.00-99.00)	97.00 (90.90-104.00)	102.00 (95.00-112.00)	110.00 (100.20-130.00)	<0.001
HbA1c (%)	5.20 (5.00-5.40)	5.40 (5.10-5.60)	5.60 (5.30-5.90)	5.80 (5.50-6.50)	<0.001
SBP (mmHg)	111.71 (105.21-120.00)	117.33 (110.00-128.00)	123.98 (115.36-135.33)	129.33 (120.00-140.67)	<0.001
DBP (mmHg)	67.25 (62.00-72.67)	70.00 (63.33-76.00)	71.88 (65.33-78.00)	70.67 (63.83-77.75)	<0.001
TG (mg/dL)	75.00 (55.00-104.00)	105.00 (74.00-152.00)	119.00 (84.00-173.00)	137.00 (97.00-195.00)	<0.001
TC (mg/dL)	181.00 (159.00-208.00)	198.00 (172.00-227.00)	200.00 (173.00-228.00)	190.00 (164.00-218.00)	<0.001
HDL-c (mg/dL)	59.00 (49.00-71.00)	53.00 (44.00-64.00)	49.00 (41.00-60.00)	45.00 (38.00-54.00)	<0.001
LDL-c (mg/dL)	103.00 (84.00-125.00)	118.00 (96.03-142.00)	121.00 (98.00-145.00)	112.02 (89.00-138.00)	<0.001
eGFR (ml/min/1.73m^2^)	110.33 (95.18-122.60)	101.93 (86.80-116.91)	94.26 (78.76-108.08)	85.30 (67.91-99.35)	<0.001
METS-VF	5.94 (5.52-6.22)	6.75 (6.61-6.87)	7.17 (7.08-7.26)	7.57 (7.46-7.72)	<0.001
Albuminuria, n%	365 (5.93%)	476 (6.36%)	721 (9.19%)	1249 (17.55%)	<0.001

PIR, Poverty-Income Ratio; CVDs, Cardiovascular Diseases; BMI, Body Mass Index; WC, Waist Circumference; FPG, Fasting Plasma Glucose; HbA1c, Hemoglobin A1c; SBP, Systolic Blood Pressure; DBP, Diastolic Blood Pressure; TG; Triglycerides; TC, Total Cholesterol, HDL-c, High-Density Lipoprotein Cholesterol, LDL-c, Low-Density Lipoprotein Cholesterol; eGFR, Estimated Glomerular Filtration Rate, METS-VF, Metabolic Score for Visceral Fat.

### 3.2 METS-VF and albuminuria risk

**Figure 3:**
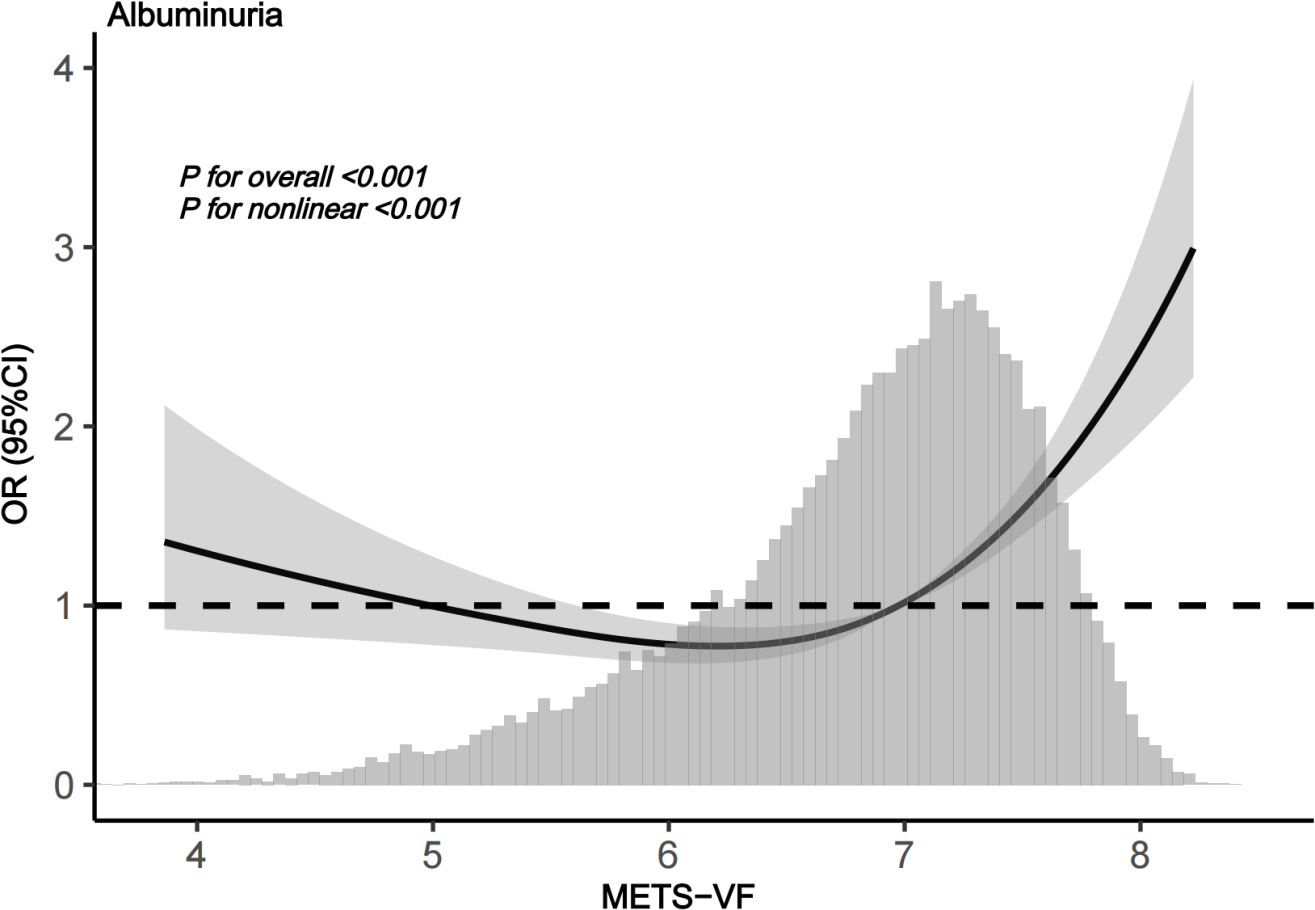
RCS analysis of METS-VF with albuminuria risk.

**Table 3** presents the logistic regression analysis results for the association between METS-V, stratified into quartiles (Q1-Q4), and the risk of albuminuria. In the unadjusted model, with Q1 as reference, the odds ratios (ORs) for Q2, Q3, and Q4 were 1.332 (95%confidence interval [CI]:1.156-1.535), 2.119 (95%CI:1.857-2.418), and 4.113 (95%CI:3.635-4.652), respectively. In Model 2, adjusted for age group, sex, ethnicity, marital status, PIR, education level, and smoking history, the ORs for Q1, Q2, Q3, and Q4 were 1.000 (reference), 1.194 (95%CI:1.033-1.380), 1.635 (95%CI: 1.424-1.878), and 2.685 (95%CI:2.341-3.079), respectively. When fully adjusting for potential confounding factors in Model 3, the ORs also maintained a comparable trend. In this fully adjusted model, the ORs for Q2, Q3, and Q4 were 1.167 (95%CI:0.991-1.374), 1.452 (95%CI:1.204-1.750), and 1.984 (95%CI:1.594-2.471), respectively, when compared to Q1 as the reference. Additionally, the analysis of METS-VF as a continuous variable also demonstrated a positive association with albuminuria risk (OR = 1.406, 95%CI:1.243-1.590) after adjusting for confounding factors. RCS analysis indicated a nonlinear, J-shaped relationship (**Figure 3**) (P for nonlinearity<0.001), with a threshold of 6.128 (**Table 4**). Below this threshold, the OR was 0.663 (95%CI:0.540-0.814); above it, the OR rose to 2.118 (95%CI:1.804-2.486).

**Table 3:** Logistic regression results of METS-VF and albuminuria risk.

Albuminuria	Model 1	Model 2	Model 3
	OR (95%CI) P value		
Continuous			
METS-VF	2.328 (2.169, 2.498) <0.001	1.789 (1.657, 1.931) <0.001	1.406 (1.243, 1.590) <0.001
Quartiles			
Q1	Reference	Reference	Reference
Q2	1.332 (1.156, 1.535) <0.001	1.194 (1.033, 1.380) 0.016	1.167 (0.991, 1.374) 0.064
Q3	2.119 (1.857, 2.418) <0.001	1.635 (1.424, 1.878) <0.001	1.452 (1.204, 1.750) <0.001
Q4	4.113 (3.635, 4.652) <0.001	2.685 (2.341, 3.079) <0.001	1.984 (1.594, 2.471) <0.001
P for trend	<0.001	<0.001	<0.001

OR: odds ratio.

95% CI: 95% confidence interval.

Model 1: non-adjusted.

Model 2: adjusted for age group (<60/≥60 years), sex, ethnicity, marital status, PIR, education level, and smoking history.

Model 3: adjusted for Model 2 + hypertension, diabetes, CVDs, BMI group (<25/25-30/≥30kg/m^2^), TC, LDL-c, and eGFR.

**Table 4:** The results of threshold effect analysis.

Model	OR (95% CI) P value
Total	1.406 (1.243, 1.590) <0.001
Breakpoint	6.128
OR1 (METS-VF<6.128)	0.663 (0.540, 0.814) <0.001
OR2 (METS-VF≥6.128)	2.118 (1.804, 2.486) <0.001
OR2/OR1	3.193 (2.403, 4.243) <0.001
P for logarithmic likelihood ratio	<0.001

OR: odds ratio.

95% CI: 95% confidence interval.

adjusted for age group (<60/≥60 years), sex, ethnicity, marital status, PIR, education level, smoking history, hypertension, diabetes, CVDs, BMI group (<25/25-30/≥30kg/m^2^), TC, LDL-c, and eGFR.

### 3.3 ROC and DCA analyses

**Figure 4:**
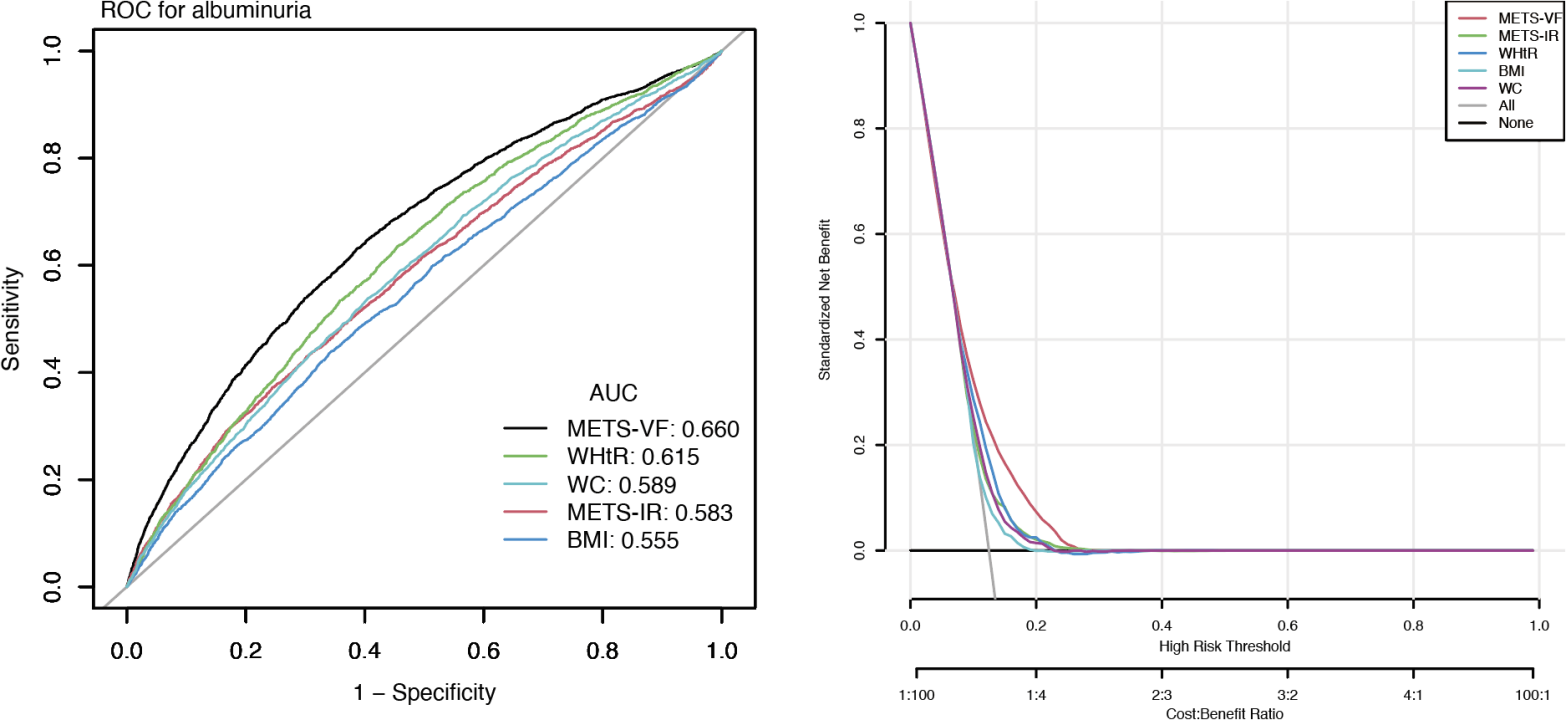
Clinical utility comparison of METS-VF (ROC and DCA analyses).

**Figure 4** displays ROC and DCA results. The area under the curve (AUC) values for METS-VF, WHtR, METS-IR, BMI, and WC were 66.0%, 61.5%, 58.3%, 55.5%, and 58.9%, respectively, indicating METS-VF’s superior discriminative ability for albuminuria risk. Additionally, DCA analysis also showed that the METS-VF model provided higher net benefit across a wider threshold probability range, suggesting greater clinical utility.

### 3.4 Subgroup and mediation analyses

**Figure 5:**
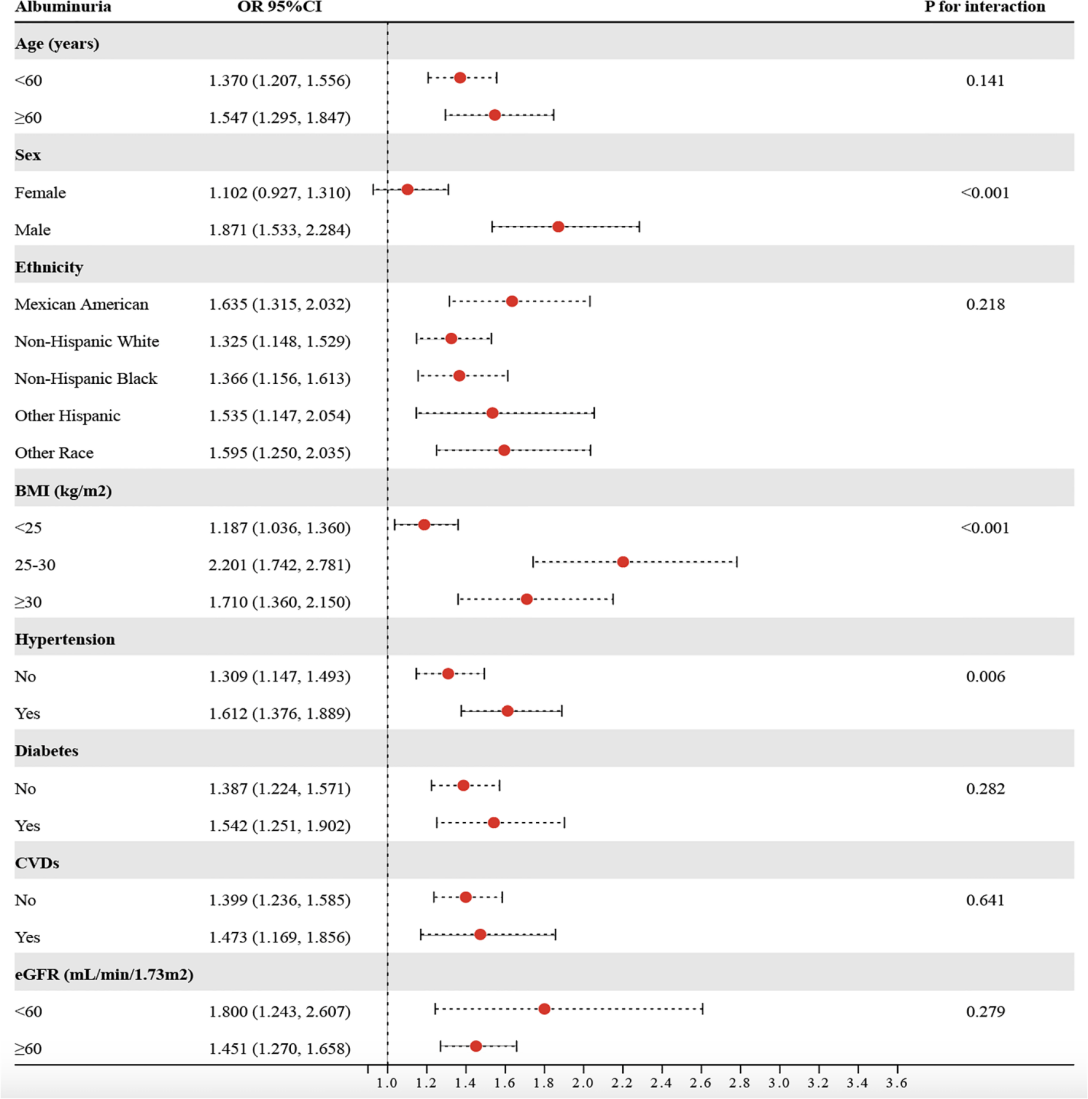
Consistency of the relationship between METS-VF and albuminuria risk across subgroups.

**Figure 6:**
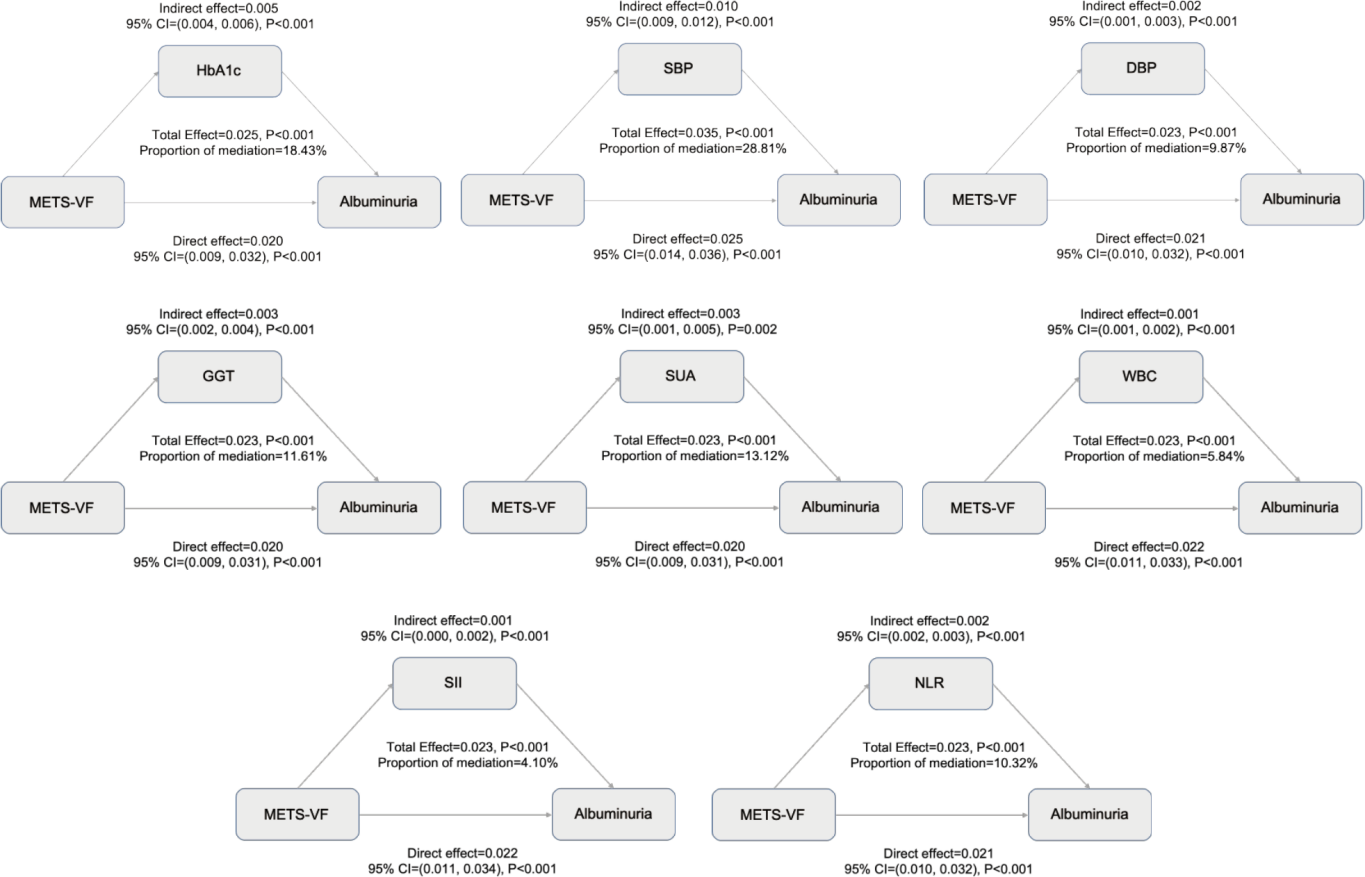
Mediating effects of HbA1c, blood pressure, oxidative stress, and inflammation on the association between METS-VF and albuminuria risk.

Based on stratified analyses, higher METS−VF was positively associated with albuminuria across age, sex, ethnicity, BMI, hypertension, diabetes, CVDs, and eGFR subgroups (**Figure 5**). By age, the ORs were 1.370 (95%CI:1.207-1.556) for <60 years and 1.547 (1.295-1.847) for ≥60 years (P for interaction = 0.141). By sex, the ORs were 1.102 (0.927-1.310) in females and 1.871 (1.533-2.284) in males (P for interaction < 0.001). By ethnicity, the ORs were 1.635 (1.315-2.032) in Mexican Americans, 1.325 (1.148-1.529) in non−Hispanic Whites, 1.366 (1.156-1.613) in non−Hispanic Blacks, 1.535 (1.147-2.054) in other Hispanics, and 1.595 (1.250-2.035) in other races (P for interaction = 0.218). By BMI, the ORs were 1.187 (1.036-1.360) for <25 kg/m², 2.201 (1.742-2.781) for 25–30 kg/m², and 1.710 (1.360-2.150) for ≥30 kg/m² (P for interaction < 0.001). By hypertension status, the ORs were 1.309 (1.147-1.493) without hypertension and 1.612 (1.376-1.889) with hypertension (P for interaction = 0.006). By diabetes status, the ORs were 1.387 (1.224-1.571) without diabetes and 1.542 (1.251-1.902) with diabetes (P for interaction = 0.282). By CVDs, the ORs were 1.399 (1.236-1.585) without CVDs and 1.473 (1.169-1.856) with CVDs (P for interaction = 0.641). By eGFR, the ORs were 1.800 (1.24-2.607) for <60 ml/min/1.73 m² and 1.451 (1.270-1.658) for ≥60 ml/min/1.73 m² (P for interaction = 0.279). Overall, associations were directionally consistent, with significant effect modification by sex, BMI, and hypertension, indicating stronger associations for males, individuals with BMI ≥25 kg/m², and those with hypertension (P for interaction < 0.05). To assess the mediating role of HbA1c, SBP, DBP, oxidative stress (GGT and SUA), and inflammation (WBC, SII, and NLR), we conducted a Sobel test, which confirmed a significant indirect effect of METS-VF on albuminuria through HbA1c (18.43%), SBP (28.81%), DBP (9.87%), GGT (11.61%), SUA (13.12%), WBC (5.84%), SII (4.10%), and NLR (10.32%) (**Figure 6**) (P<0.001).

## 4 Discussion

To our knowledge, this is the first study to investigate the link between METS-VF and albuminuria in a nationally representative cohort. METS-VF is an independent risk factor for albuminuria and may serve as a direct and valuable epidemiological tool for assessing visceral fat’s contribution to albuminuria risk.

Epidemiological research has long faced the “obesity paradox,” where the complexity of anthropometric data complicates precise risk identification (25, 26). Visceral fat, a key driver of metabolic dysfunction, promotes systemic inflammation and insulin resistance through pro-inflammatory cytokines (e.g., IL-6, TNF-α), leading to glomerular filtration barrier dysfunction and albuminuria (27–29). Excess free fatty acids and lipotoxicity further exacerbate renal cell and microvascular injury (30, 31). Our study revealed that HbA1c and blood pressure mediate the relationship between the METS-VF and albuminuria. Elevated METS-VF reflects visceral fat accumulation, which worsens insulin resistance and chronic inflammation, thereby increasing HbA1c levels (32, 33). Prolonged hyperglycemia damages the glomerulus via oxidative stress and advanced glycation end products (AGEs) (34, 35). Additionally, visceral fat activates the renin-angiotensin-aldosterone system (RAAS), raising blood pressure and inducing glomerular hyperfiltration, which accelerates renal injury (36, 37). Therefore, strategies targeting visceral fat may improve metabolic health and reduce albuminuria risk. METS-VF provides a simple, noninvasive, and cost-effective tool for assessing visceral fat and associated cardiometabolic risks (14). ROC and DCA confirmed its superior discriminative power and clinical utility compared to conventional anthropometric measures. In ROC analysis, the AUC of METS−VF for identifying proteinuria was approximately 0.66, consistent with the multifactorial etiology of albuminuria, whereby a single metric is unlikely to achieve high diagnostic performance. These findings underscore the need for multi−marker models; integrating oxidative stress and inflammatory markers with METS−VF may improve predictive performance and clinical net benefit.

Our study further revealed a characteristic J-shaped relationship between METS-VF and albuminuria risk. At lower METS-VF levels, the risk of albuminuria remained relatively stable or even decreased, possibly due to the preserved compensatory capacity of VAT in early stages (38–40). When METS-VF exceeds a threshold, albuminuria risk rises sharply due to synergistic effects of visceral fat-induced lipotoxicity, chronic inflammation, and insulin resistance, which collectively worsen metabolic dysfunction and organ damage (39, 41). The METS-VF threshold range may reflect an optimal metabolic balance of visceral fat, serving as a valuable clinical indicator for metabolic health (41, 42). It is important to emphasize that UACR is a mature, rapid, and guideline−endorsed test for detecting albuminuria, and our study does not aim to replace it. Instead, METS−VF integrates multidimensional information-metabolic profile, lipids, and anthropometrics-to provide a system−level risk assessment as a risk−stratification tool; in individuals without albuminuria, higher METS−VF may indicate earlier cardiometabolic–renal susceptibility and thus a longer window for preventive intervention, whereas in those with albuminuria it can offer mechanistic context-such as the burden of visceral adiposity and insulin resistance-to inform individualized risk modification and optimization of lifestyle and metabolic targets.

This study also has some limitations. The cross-sectional design precludes causal inference, necessitating prospective validation. Residual confounding may persist despite multivariable adjustment-given the relatively limited information available in NHANES-such as incomplete detail on diet, exercise habits, and medication use. METS-VF relies on fasting glucose and lipids, which may also fluctuate. Our mediation analysis was exploratory and hypothesis−generating; it assumed a directional exposure-mediator-outcome sequence that cannot be confirmed in cross−sectional data, and reverse or bidirectional relationships cannot be excluded. Additionally, our mediation analysis focused on common biomarkers and did not assess other pathways, such as lipotoxicity, due to suitable surrogate indicators were unavailable in our dataset. Threshold applicability across populations needs further validation. Future studies should combine multicenter cohorts and broader biomarkers to assess METS-VF’s value.

## 5 Conclusion

In this cross-sectional study, higher METS−VF was associated with higher prevalence of albuminuria, with a J−shaped relationship. However, these findings reflect associations and do not establish causality or predictive value. Prospective longitudinal studies are needed to assess temporality and prediction, clarify underlying mechanisms, and evaluate clinical utility in real−world settings.

## Data Availability

Publicly available datasets were analyzed in this study. This data can be found here: https://wwwn.cdc.gov/nchs/nhanes.
